# Modulation of Gut Mycobiome and Serum Metabolome by a MUFA-Rich Diet in Sprague Dawley Rats Fed a High-Fructose, High-Fat Diet

**DOI:** 10.3390/foods14030506

**Published:** 2025-02-05

**Authors:** Zhihao Zhao, Lihuang Zhong, Jiajin Wu, Guangzhen Zeng, Songbin Liu, Yuanyuan Deng, Yan Zhang, Xiaojun Tang, Mingwei Zhang

**Affiliations:** 1 Sericultural & Agri-Food Research Institute, Guangdong Academy of Agricultural Sciences/Key Laboratory of Functional Foods, Ministry of Agriculture and Rural Affairs/Guangdong Key Laboratory of Agricultural Products Processing, Guangzhou 510610, China; zhaozhihao1991@163.com (Z.Z.); zhonglihuang1993@163.com (L.Z.); zenggz2022@163.com (G.Z.); gmp.gap.iso@gmail.com (S.L.); yuanyuan_deng@yeah.net (Y.D.); zhang__yan_@126.com (Y.Z.); xjtang66@163.com (X.T.); 2 School of Medicine, Southeast University, Nanjing 210009, China; dr2017jjw@163.com

**Keywords:** dietary fatty acids, oleic acid, high-fat, high-fructose diet, gut fungi, bile acid metabolism, CoA biosynthesis

## Abstract

The intake of oleic acid-rich fats, a hallmark of the Mediterranean diet, has well-documented beneficial effects on human metabolic health. One of the key mechanisms underlying these effects is the regulation of gut microbiota structure and function. However, most existing studies focus on gut bacteria, while gut fungi, as a vital component of the gut microbiota, remain largely unexplored. This study compared the effects of regular peanut oil (PO) and high-oleic acid peanut oil (HOPO) on the gut mycobiome and serum metabolome employing ITS high-throughput sequencing and UPLC-MS/MS metabolomics to explore how dietary fatty acid composition influences gut microecology. Both HOPO and PO effectively reversed high-fat, high-fructose diet (HFFD)-induced reductions in gut fungal diversity, with HOPO showing superior efficacy in restoring gut microbiome balance, as reflected by an improved fungal-to-bacterial diversity ratio and reduced abundance of pathogenic fungi such as *Aspergillus*, *Penicillium*, and *Candida*. Furthermore, HOPO demonstrated a greater ability to normalize serum bile acid levels, including taurochenodesoxycholic acid, and to reverse elevated pantothenol levels, suggesting its potential role in maintaining bile acid metabolism and CoA biosynthesis. In summary, HOPO is more effective than PO in maintaining the normal structure and function of gut mycobiome in HFFD-fed SD rats.

## 1. Introduction

Fats are one of the three primary macronutrients that provide energy for the human body. Beyond serving as a major energy source, they also supply essential fatty acids, which are critical for maintaining normal physiological functions and overall health. Fats also play indispensable roles in supporting cellular structure, hormone synthesis, and the absorption of fat-soluble vitamins, underscoring their fundamental contribution to human physiology [1]. However, dietary fats represent a paradox in human health. While they are essential for maintaining physiological functions, excessive intake of dietary fats, particularly those high in saturated fatty acids, has been identified as a significant contributor to the development of various chronic diseases [2]. These include obesity, cardiovascular diseases, non-alcoholic fatty liver disease (NAFLD), and other related conditions. High consumption of saturated fats can lead to dyslipidemia, systemic inflammation, and insulin resistance, which are key risk factors for these conditions [3,4]. Thus, both the type and quantity of dietary fat intake are critical determinants of their impact on health. The underlying mechanisms involve the cytosolic ectopic accumulation of fatty acids [5], endothelial dysfunction [6], mitochondrial dysfunction [7], endoplasmic reticulum stress [8], and gut microbiome dysbiosis [9].

The gut microbiome, a diverse and dynamic community of trillions of microorganisms residing in the gastrointestinal tract, is integral to maintaining host health [10]. They play a key role in essential physiological processes, including nutrient metabolism, vitamin synthesis, and the fermentation of dietary fibers into short-chain fatty acids [11], which regulate host metabolic and immune functions through GPR41 and GPR43 [12]. Additionally, the gut microbiome plays a pivotal role in immune system development, defense against pathogens, and the maintenance of intestinal epithelial barrier integrity [13,14]. Dysbiosis, or an imbalance in the gut microbiome, has been strongly associated with various health conditions, including obesity, metabolic syndrome, diabetes, inflammatory bowel disease, and even neurological disorders [15,16]. Recent studies have highlighted the potential of modulating the gut microbiome through dietary interventions, probiotics, and prebiotics as a promising strategy for preventing and managing these diseases [17,18]. Therefore, understanding the intricate relationship between the gut microbiome and human health is essential for developing novel therapeutic approaches.

Previous studies on the gut microbiome have primarily focused on bacteria, which dominate in terms of abundance. However, recent research has revealed that symbiotic fungi, although low in absolute numbers, are highly diverse and play a significant role in human health and disease. Unlike bacterial cells, fungal cells are larger, more complex, and possess unique metabolic pathways, enabling them to adapt to diverse environmental conditions and interact with their host in distinct ways [19]. Gut fungi can influence host health both independently and through interactions with gut bacteria. These interactions may involve competition for nutrients, co-metabolism of dietary components, or modulation of the host immune system [20]. Certain fungal species can enhance bacterial colonization or biofilm formation, while others may inhibit bacterial growth through the production of antifungal compounds. For instance, *Saccharomyces boulardii*, a yeast widely studied as a potential probiotic, has demonstrated protective effects against numerous bacterial gastrointestinal pathogens, such as *Clostridium difficile*, *Helicobacter pylori*, *Vibrio cholerae*, and *Escherichia coli* [20]. These interactions highlight the antipathogenic potential of commensal fungi, while commensal bacteria can also protect against pathogenic fungi. Such interkingdom interactions are critical for maintaining gut homeostasis and shaping the overall microbial community structure [21]. The impact of gut fungi on human health is multifaceted. On the one hand, commensal fungi contribute to the digestion of complex carbohydrates, the production of bioactive metabolites, and the regulation of immune responses. On the other hand, dysbiosis of the fungal community has been associated with various diseases, including inflammatory bowel disease, metabolic disorders, and allergies [19]. These findings underscore the need for a deeper understanding of the fungal microbiota and its role in health and disease.

Over the past few decades, studies on the Mediterranean diet have increasingly emphasized the health-promoting effects of olive oil and other oils rich in oleic acid, including their ability to prevent obesity, regulate glucose and lipid metabolism, and provide antioxidant and anti-inflammatory benefits [22,23]. As a result, enhancing oleic acid content has become an important objective in the breeding of new oil crop varieties. High-oleic acid peanuts are selectively bred peanut varieties with significantly higher oleic acid content compared to regular peanuts, typically exceeding 75%. They exhibit enhanced oxidative stability and extended shelf life, while also offering significant benefits for metabolic health, making them increasingly valued in recent years. Our previous study demonstrated that high-oleic acid peanut oil (HOPO) has a more pronounced effect than regular peanut oil (PO) in mitigating insulin resistance induced by a high-fat/high-fructose diet, likely through its modulation of gut bacterial structure and function [24]. However, the effects of high-oleic acid and regular peanut oils on the structure and functionality of the gut mycobiome remain unclear.

This study further conducted a comparative analysis of the effects of PO and HOPO on regulating the gut mycobiome and serum metabolome. Both HOPO and PO reversed HFFD-induced reductions in fungal diversity, with HOPO showing greater efficacy in restoring microbiome balance by improving the fungal diversity/bacterial diversity ratio and reducing pathogenic fungi (*Aspergillus*, *Penicillium*, *Candida*). HOPO also more effectively normalized serum bile acid levels (e.g., taurochenodesoxycholic acid) and reversed elevated pantothenol levels, highlighting its potential role in supporting bile acid metabolism and CoA biosynthesis, thereby maintaining normal glucose and lipid metabolism in rats. In summary, HOPO is more effective than PO in maintaining the normal structure and function of the gut mycobiome in HFFD-fed SD rats. This study offers deeper insights into how dietary fatty acid composition influences gut microecology.

## 2. Materials and Methods

### 2.1. Materials

The Luhua Group (Laiyang, China) supplied the PO and HOPO, which feature an oleic acid content of 43.23% with an oleic acid to linoleic acid ratio of 1.27, and 76.83% with a ratio of 11.48, respectively. Fructose was sourced from Baolingbao Biology Co., Ltd. located in Binzhou, China.

### 2.2. Animals and Dietary Intervention Design

Forty male Sprague Dawley rats (6–7 weeks old, 180 ± 20 g, SPF grade) were sourced from Guangdong Medical Laboratory Animal Center, Foshan, China. Following a 1-week acclimation period, the rats were divided into four groups at random (n = 10) for an 18-week dietary intervention. The normal control (NC) group was fed a standard chow diet with distilled water. The model (M) group, the high-oleic acid peanut oil (HOPO) group, and the regular peanut oil (PO) group received a diet with 45% high-fat content and 10% fructose water. Table 1 provides a detailed overview of the nutrient profiles and fat sources in the test diets. The rats were maintained in a well-ventilated environment with regulated conditions, featuring a 12 h light/dark cycle, a consistent temperature of 23 ± 2 °C, and relative humidity around 55 ± 5%. Fresh fecal samples were gathered two days prior to the execution and immediately stored in a refrigerator at −80 °C. After the dietary intervention concluded, the rats were sacrificed following a 10 h fast. Blood was drawn and subsequently centrifuged at 4 °C at 2000× *g* for 15 min to obtain serum samples. The protocol for the animal study was approved by the Animal Ethical and Welfare Committee of the Sericultural & Agri-Food Research Institute, Guangdong Academy of Agricultural Sciences (approval code: 2022-SC-07, approval date: 19 January 2022).

### 2.3. Gut Mycobiome Community Analysis

The community structure of the gut mycobiome of the rats was analyzed using high-throughput sequencing of ITS sequences. The PowerSoil^®^ DNA Isolation Kit (MoBio, Carlsbad, CA, USA) was employed to extract genomic DNA from the total microbial community in fecal samples, following the manufacturer’s instructions. The ITS sequences were amplified with the primers ITS1F (5′-CTTGGTCATTTAGAGGAAGTAA-3′) and ITS4R (5′-TCCTCCGCTTATTGATATGC-3′). The high-quality purified PCR products were sequenced using a PacBio sequencing platform (Pacific Biosciences, Menlo Park, CA, USA). CCS sequences were identified from raw subreads using SMRT Link v8.0, applying criteria of a minimum of 5 passes and a minimum predicted accuracy of 0.9. Barcode identification and length filtering of CCS sequences were performed using lima v1.7.0 and cutadapt v2.7. Following this, chimeras were eliminated using UCHIME v8.1, resulting in the acquisition of optimized CCS reads. Indices related to microbial community analysis, including operational taxonomic units (OTUs) richness, alpha diversity indices, beta diversity, and LEfSe analysis, were examined using Usearch v10.0, LEfSe software (https://huttenhower.sph.harvard.edu/lefse/), and QIIME2 (v2020.6) on the BMK Cloud platform (www.biocloud.net).

### 2.4. Serum Metabolome Analysis

A 50 μL serum sample was extracted for 3 min under vortexing, using 300 μL of a methanol and acetonitrile solution (8:2, *V*/*V*) containing an internal standard. We centrifuged the mixture at 12,000 rpm and 4 °C for 10 min to separate and collect the supernatant. Then, we performed another centrifugation at 12,000 rpm and 4 °C for an additional 3 min. The supernatant underwent analysis using a UPLC system (Shim-pack UFLC SHIMADZU CBM30A) paired with a tandem mass spectrometer (Applied Biosystems 4500 QTRAP, Waltham, MA, USA). The analysis used a C18 chromatographic column (1.8 μm, 2.1 × 100 mm) and employed solvent A (0.1% formic acid in ultrapure water), along with solvent B (0.1% formic acid in acetonitrile), as the mobile phase. The chromatographic conditions were set as follows: the elution gradient started at 0 min with a water/acetonitrile ratio of 95:5 (*V*/*V*), was changed to 10:90 (*V*/*V*) at 11.0 min, maintained at 10:90 (*V*/*V*) until 12.0 min, then reverted to 95:5 (*V*/*V*) at 12.1 min, and remained at 95:5 (*V*/*V*) until 14.0 min. The flow rate was maintained at 0.4 mL/min, with a column temperature of 40 °C, and an injection volume of 2 μL. The ESI source operated with a source temperature of 500 °C. Ion spray voltages were set at 5.5 kV for positive mode and −4.5 kV for negative mode. The pressures were set at 55 psi for ion source gas I, 60 psi for gas II, and 25 psi for the curtain gas, respectively. The qualitative analysis utilized the laboratory’s custom-built targeted standard database, developed using retention time, ion pair details, and secondary spectral information.

In the two-group analysis, metabolites were considered significantly regulated if they had a VIP score of ≥1 and a Student’s *t*-test *p*-value of <0.05. These metabolites were annotated via the KEGG Compound database (http://www.kegg.jp/kegg/compound/, accessed on 2 February 2025) and subsequently mapped to the KEGG Pathway database (http://www.kegg.jp/kegg/pathway.html, accessed on 2 February 2025). Pathways containing these significantly regulated metabolites were identified and analyzed using Metabolite Sets Enrichment Analysis (MSEA). The significance of the pathways was evaluated using p-values obtained from the hypergeometric test.

### 2.5. Statistical Analysis

All data are expressed as mean ± standard deviation (SD). Statistical analyses were conducted using SPSS software (version 22.0), with significance determined by Duncan’s multiple range test at a *p*-value < 0.05. Graphic analyses were performed using Origin software (2019b).

## 3. Results

### 3.1. α-Diversity of Gut Mycobiome

**Figure 1:**
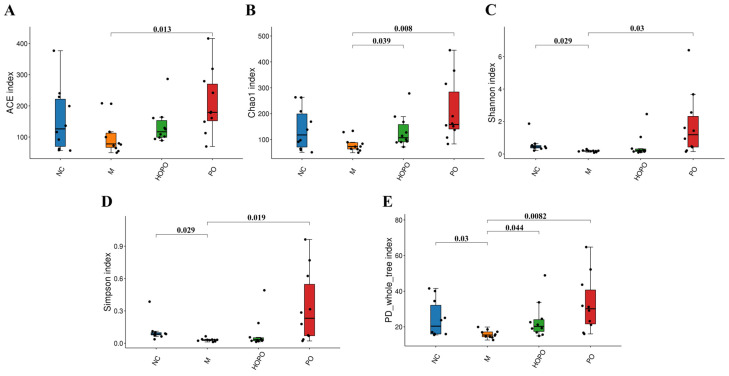
Alpha diversity of gut mycobiome in rats. (**A**) ACE index; (**B**) Chao1 index; (**C**) Shannon index; (**D**) Simpson index; (**E**) PD_whole_tree index. Numbers indicate *p* values between two groups. n = 10 for each group.

The alpha diversity of gut mycobiome within each group was assessed using various indices, including ACE, Chao1, Shannon, Simpson, and the PD_whole_tree index (Figure 1). In the four groups of rats, the five alpha diversity indices for gut mycobiome exhibited a consistent trend: the PO group showed the highest values, followed by the NC group, then the HOPO group, and the M group displayed the lowest values. Compared to the NC group, the M group showed a significant decrease in the Shannon and Simpson indices (*p* < 0.05), which are based on community richness and community evenness, as well as in the PD_whole_tree index (*p* < 0.05), which is based on a phylogenetic tree. However, there were no significant differences in the ACE and Chao1 indices, which are based solely on species richness. This indicates that HFFD induced a reduction in both community richness and evenness of gut mycobiome in rats, particularly affecting community evenness. Compared to the M group, the PO group showed significant increases across all five indices (*p* < 0.05). In contrast, the HOPO group showed significant differences solely in the Chao1 and PD_whole_tree indices (*p* < 0.05). This suggests that both PO and HOPO can enhance the alpha diversity of gut mycobiome in rats fed with HFFD. However, compared to PO, HOPO is less effective in increasing the alpha diversity of gut mycobiome.

### 3.2. β-Diversity of Gut Mycobiome

**Figure 2:**
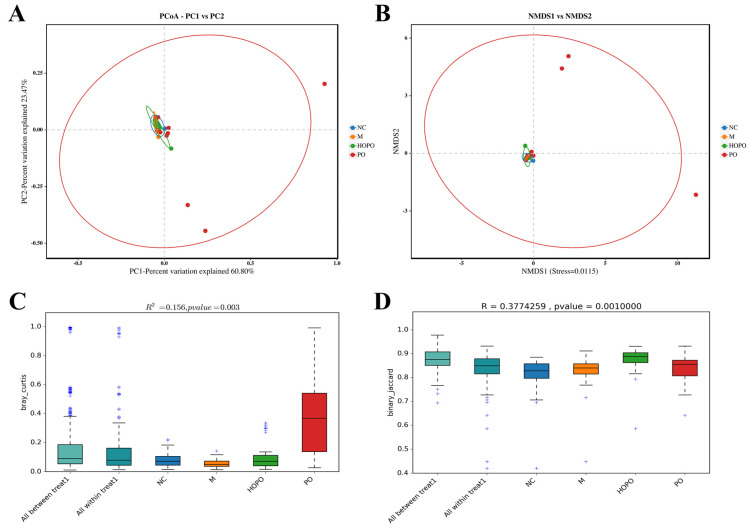
Beta diversity of gut mycobiome in rats. (**A**) PCoA based on Bray–Curtis. (**B**) NMDS based on Bray–Curtis. (**C**) PERMANOVA based on Bray–Curtis. (**D**) Anosim based on Binary–Jaccard. n = 10 for each group.

Various beta diversity indices, such as PCoA, NMDS, PERMANOVA, and ANOSIM, were employed to assess the structural differences in the gut mycobiome across different groups. In the PCoA and NMDS scatter plots based on Bray–Curtis (Figure 2A,B), the sample distributions of the NC, M, and HOPO groups appear relatively concentrated, whereas the PO group shows a more dispersed distribution. As shown in Figure 2C, the PERMANOVA analysis indicated that different diets explained 15.60% of the variation in the gut mycobiome community structure in rats (R^2^ = 0.156, *p* < 0.01). ANOSIM analysis demonstrated that a moderate R value of approximately 0.38, coupled with a highly significant *p*-value (<0.01), indicates that diet is a significant factor influencing the gut mycobiome communities in rats (Figure 2D). Although the separation is not particularly strong, the statistical significance indicates that dietary differences have indeed led to significant changes in the fungal community structure. These results highlight the role of dietary fat in shaping gut mycobiome composition.

### 3.3. Composition of Gut Mycobiome

**Figure 3:**
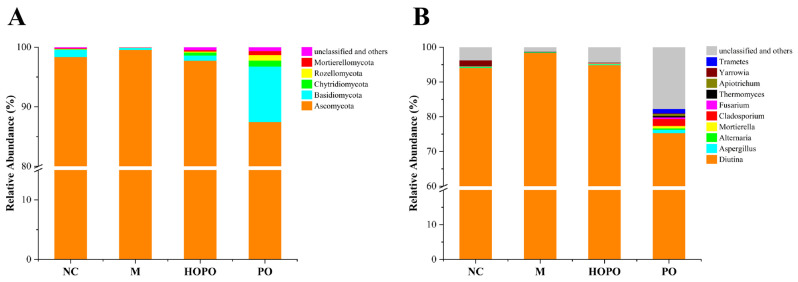
Composition of gut mycobiome in rats. (**A**) Relative abundance at phylum level. (**B**) Relative abundance at genus level. n = 10 for each group.

Figure 3A illustrates the composition of the gut mycobiome in rats at the phylum level. *Ascomycota* was the dominant phylum in four groups, with a total relative abundance ranging from 87.47% to 99.55%. Compared to the NC group, the M group showed a higher relative abundance of *Ascomycota*, accompanied by a decrease in the relative abundances of *Basidiomycota*, *Chytridiomycota*, *Rozellomycota*, and *Mortierellomycota*. However, these changes were reversed in the HOPO and PO groups compared to the M group. Notably, the PO group displayed the lowest abundance of *Ascomycota* among the four groups, while the relative abundances of other fungal phyla were the highest. This suggests that PO has a more comprehensive regulatory effect on the gut fungal community, potentially improving gut ecological balance by inhibiting the overgrowth of *Ascomycota* and promoting the recovery of other fungal phyla.

Figure 3B displays the top ten genera identified in the gut mycobiome of rats. *Diutina* was the dominant genera in four groups, with a total relative abundance ranging from 75.30% to 98.43%. Compared to the NC group, the M group showed an increased relative abundance of *Diutina*, along with a reduction in the abundances of *Alternaria*, *Apiotrichum*, *Aspergillus*, *Cladosporium*, *Fusarium*, *Mortierella*, *Thermomyces*, and *Yarrowia*. However, these changes were reversed in the PO group, while all changes except those involving *Thermomyces* and *Yarrowia* were reversed in the HOPO group compared to the M group. Notably, the PO group exhibited the lowest relative abundance of *Diutina* among the four groups, while the abundances of the other top ten fungal genera, except for *Yarrowia*, were the highest. This further supports the role of PO and HOPO in restoring the diversity of the gut fungal community, with PO showing a more pronounced effect.

### 3.4. LEfSe Analysis of Gut Mycobiome

**Figure 4:**
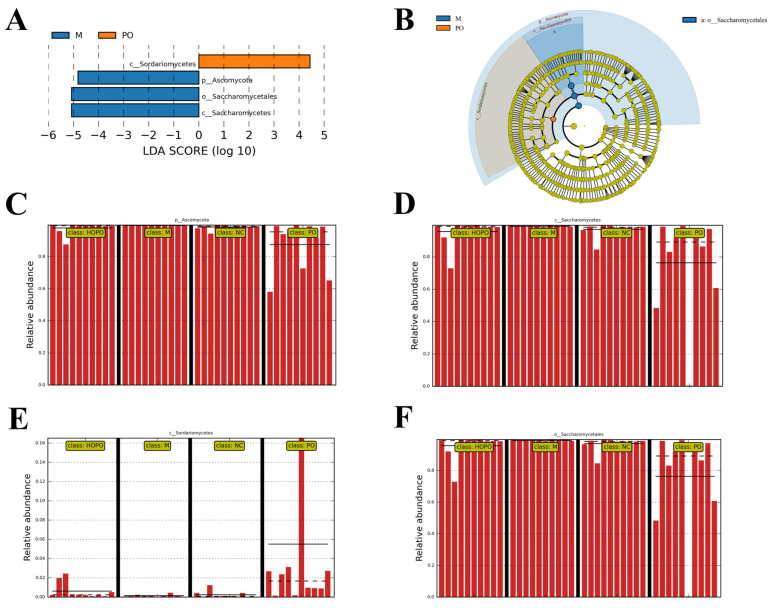
LEfSe analysis of gut mycobiome in rats. (**A**) LDA distribution displays species with significant abundance (LDA > 4) across different groups. (**B**) Hierarchical tree for different groups. The outward-radiating circles depict taxonomic levels ranging from phylum to species. The small circle at each specific level indicates a classification, with its diameter reflecting the species’ relative abundance. (**C**–**F**) Relative abundance of species with significant abundance across different groups. The solid and dashed lines represent the mean and median relative abundances, respectively. n = 10 for each group.

LEfSe (Linear Discriminant Analysis Effect Size) is a statistical tool designed to identify biomarkers that exhibit significant differences between groups in high-dimensional datasets. LEfSe combines non-parametric statistical tests with Linear Discriminant Analysis (LDA) to evaluate the significance and effect size of features, making it ideal for analyzing complex biological data, such as microbiome studies. Figure 4 presents the LEfSe analysis results of the gut mycobiome across various groups, using an LDA threshold of 4.0. Four fungal taxa, all belonging to *Ascomycota*, exhibited significant differences among the groups. Of these, three fungal taxa—*Ascomycota*, *Saccharomycetales*, and *Saccharomycetes*—were more abundant in the M group, while one fungal taxon, *Sordariomycetes*, was more abundant in the PO group (Figure 4A,B). No specific dominant fungal taxa were observed in either the NC or HOPO groups. The abundances of the four significantly different fungal taxa—*Ascomycota*, *Saccharomycetales*, *Saccharomycetes*, and *Sordariomycetes*—across the groups are shown in Figure 4C. The M group, compared to the NC group, exhibited a higher relative abundance of *Ascomycota*, *Saccharomycetales*, and *Saccharomycetes*, while showing a lower abundance of *Sordariomycetes*. These alterations were reversed in the HOPO and PO groups when compared to the M group. Notably, the M group exhibited the highest relative abundance of *Ascomycota*, *Saccharomycetales*, and *Saccharomycetes* among the four groups, while the relative abundance of *Sordariomycetes* was the lowest. In contrast, the PO group displayed the lowest relative abundance of *Ascomycota*, *Saccharomycetales*, and *Saccharomycetes*, but the highest relative abundance of *Sordariomycetes* among the four groups. These results further support the significant role of PO in restoring the ecological balance of the gut fungal community.

### 3.5. Correlation Network Analysis of Gut Mycobiome

**Figure 5:**
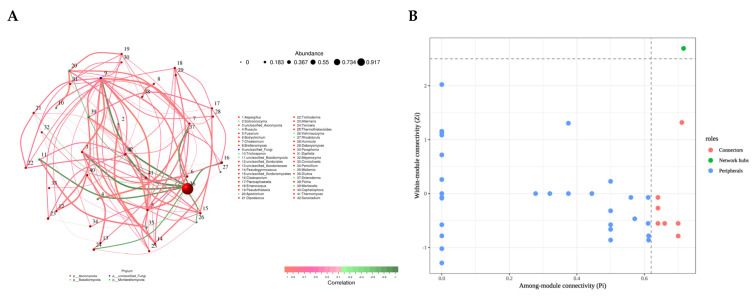
(**A**) Correlation network of gut mycobiome at genus level in rats. Circles represent species, with circle size indicating the average abundance of each species. Lines represent correlations between species, with line thickness indicating the strength of the correlation. Red lines represent positive correlations, and green lines represent negative correlations. (**B**) Zi-Pi distribution plot of nodes. n = 10 for each group.

The gut mycobiome functions as a complex micro-ecosystem with intricate interactions. Analyzing the co-occurrence networks of fungal taxa provides valuable insights into the modulation of gut mycobiome interactions in rats. The visualization of the resulting network diagram is presented in Figure 5A. *Diutina* was found to be correlated with the following fungal taxa: *Aspergillus*, *unclassified_Ascomycota*, *unclassified_Fungi*, *unclassified_Basidiomycota*, *Cladosporium*, *Chaetomium*, *Yarrowia*, *Vishniacozyma*, *Apiotrichum*, *Dipodascus*, *Wallemia*, *unclassified_Sordariomycetes*, *Plectosphaerella*, and *Alternaria*. Notably, all of these correlations were negative. *Diutina*, a fungal genus within the phylum *Ascomycota*, is consistent with the fungal abundance composition observed at both the phylum and genus levels in the gut. Among these fungal taxa, two pairs exhibited extremely strong correlations. *Aspergillus* and *Diutina* showed a strong negative correlation (R < −0.70), while *Solicoccozyma* and *Pseudogymnoascus* exhibited a strong positive correlation (R > 0.70). Further analysis of node types was conducted for these fungal taxa. As shown in Figure 5B, among the 42 nodes, a single node was recognized as a network hub (zi > 2.50, Pi > 0.62), 7 nodes were identified as connectors (zi ≤ 2.50, Pi > 0.62), and the remaining nodes were classified as peripheral nodes (zi ≤ 2.50, Pi ≤ 0.62). The fungal taxon identified as the network hub was *unclassified_Fungi*, while the seven fungal taxa identified as connectors included *Aspergillus*, *unclassified_Basidiomycota*, *Alternaria*, *Vishniacozyma*, *Penicillium*, *Pseudogymnoascus*, and *Thermothielavioides*. These fungal taxa may play a more significant role in shaping the structure and function of the fungal community in the rat gut compared to other fungal taxa.

### 3.6. Analysis of Serum Metabolome Profile

**Figure 6:**
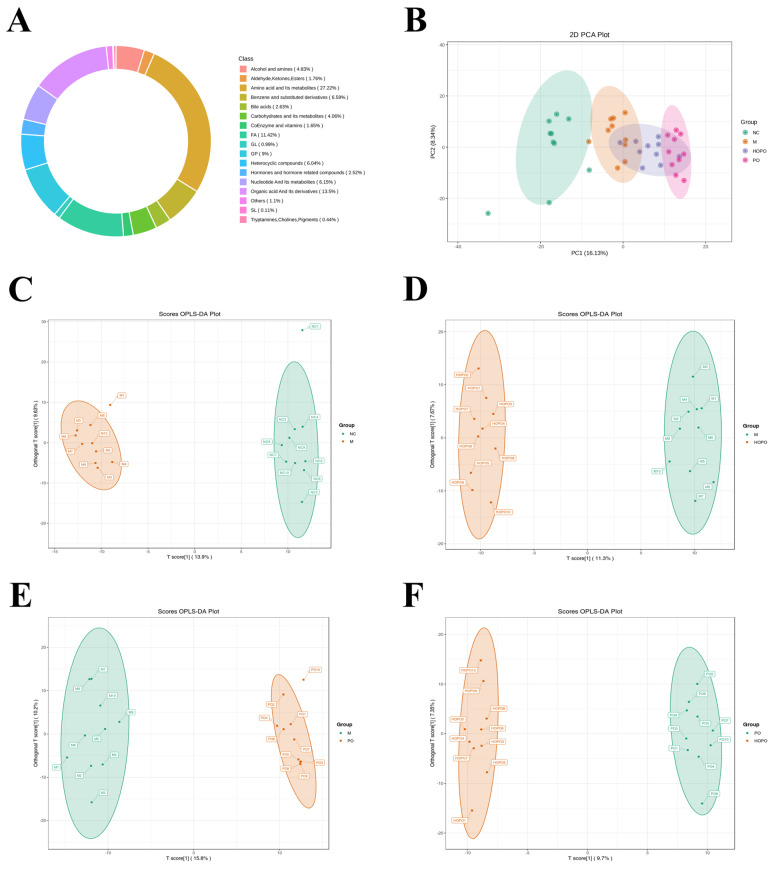
Serum metabolome profile of rats. (**A**) Circular chart of metabolite category composition. (**B**) PCA score plot of samples from each group. (**C**–**F**) OPLS-DA score plots comparing NC vs. M, M vs. HOPO, M vs. PO, and PO vs. HOPO groups. n = 10 for each group.

The metabolites detected in rat serum were classified into 17 categories, among which amino acids and their metabolites represented the class with the highest relative abundance, accounting for 27.22% (Figure 6A). PCA analysis revealed distinct clustering patterns among the four groups (NC, M, HOPO, and PO), emphasizing differences in their overall metabolic profiles (Figure 6B). Furthermore, OPLS-DA analysis demonstrated clear separations between the two groups, indicating distinct metabolic compositions (Figure 6C–F). As shown in Figure S1, the OPLS-DA models were validated using permutation tests. All four models exhibited R^2^Y values exceeding 0.98 (*p* < 0.005), demonstrating excellent explanatory power. Additionally, the Q^2^ values for all four models were greater than 0.74 (*p* < 0.005). In addition, all four models had Q^2^ values exceeding 0.74 (*p* < 0.005), demonstrating strong predictive efficiency. Together, these findings confirm that the OPLS-DA models effectively identified distinct metabolites across the groups, revealing significant differences in serum metabolite profiles. This highlights the metabolic impact of different diets, with the high-oleic acid content in peanut oil, inducing notable alterations in the serum metabolome of rats fed a HFFD.

### 3.7. Metabolic Biomarkers and Pathway Enrichment of Serum Metabolome

**Figure 7:**
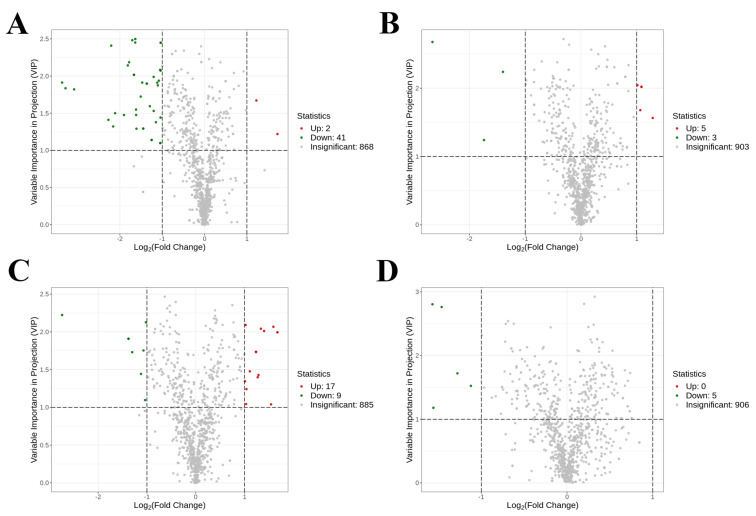
Volcano plots of serum metabolome. (**A**) Volcano plots comparing NC and M groups. (**B**) Volcano plots comparing M and PO groups. (**C**) Volcano plots comparing M and PO groups. (**D**) Volcano plots comparing PO and HOPO groups. n = 10 for each group.

Volcano plots were constructed to visualize the differential metabolites among the groups, utilizing a combined screening approach based on VIP values (VIP > 1) and fold changes (fold change > 2 or <0.5). As depicted in Figure 7A–D, a total of 43, 8, 26, and 5 serum biomarkers were detected between the NC vs. M, M vs. HOPO, M vs. PO, and PO vs. HOPO groups, respectively.

**Figure 8:**
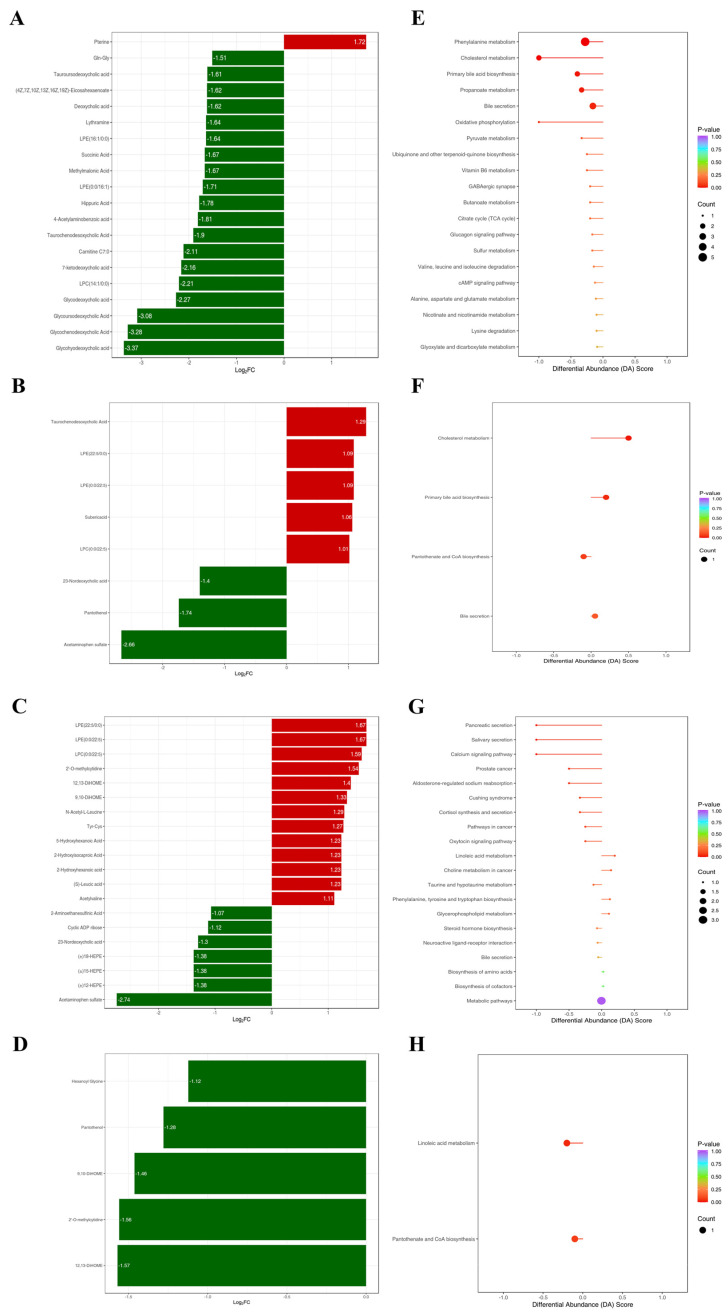
Serum metabolic biomarkers and pathway enrichment analysis. (**A**–**D**) The top 20 metabolites with the largest fold changes between the NC and M groups, M and HOPO groups, M and PO groups, and PO and HOPO groups, respectively. If fewer than 20 differential metabolites are identified, all are shown. (**E**–**H**) Differential abundance scores based on KEGG pathway analysis for the NC vs. M, M vs. HOPO, M vs. PO, and PO vs. HOPO groups, respectively. n = 10 for each group.

Among the top 20 differential metabolites ranked by fold change, the M group exhibited a significantly higher relative level of pterine compared to the NC group. In contrast, the relative levels of Gln-Gly, tauroursodeoxycholic acid, (4Z,7Z,10Z,13Z,16Z,19Z)-eicosahexaenoate, deoxycholic acid, lythramine, LPE (16:1/0:0), succinic acid, methylmalonic acid, LPE (0:0/16:1), hippuric acid, 4-acetylaminobenzoic acid, taurochenodesoxycholic acid, carnitine C7:0, 7-ketodeoxycholic acid, LPC (14:1/0:0), glycodeoxycholic acid, glycoursodeoxycholic acid, glycochenodeoxycholic acid, and glycohyodeoxycholic acid were significantly decreased (Figure 8A). The identified biomarkers distinguishing the NC and M groups were primarily associated with pathways involved in energy metabolism, amino acid metabolism, lipid metabolism, and vitamin and cofactor metabolism (Figure 8E).

Compared to the M group, the HOPO group exhibited a significantly decreased relative level of 23-nordeoxycholic acid, pantothenol, and acetaminophen sulfate among the 8 differential metabolites. In contrast, the relative levels of taurochenodesoxycholic acid, LPE (22:5/0:0), LPE (0:0/22:5), suberic acid, and LPC (0:0/22:5) were significantly increased (Figure 8B). The identified biomarkers distinguishing the M and HOPO groups were primarily associated with four pathways of cholesterol metabolism, primary bile acid biosynthesis, pantothenate and CoA biosynthesis, and bile secretion (Figure 8F).

Compared to the M group, the PO group exhibited a significantly increased relative level of LPE (22:5/0:0), LPE (0:0/22:5), LPC (0:0/22:5), 2′-O-methylcytidine, 12,13-DiHOME, 9,10-DiHOME, N-acetyl-L-leucine, Tyr-Cys, 5-hydroxyhexanoic acid, 2-hydroxyisocaproic acid, 2-hydroxyhexanoic acid, (S)-leucic acid, and acetylvaline among the top 20 differential metabolites ranked by fold change. In contrast, the relative levels of 2-aminoethanesulfinic acid, cyclic ADP ribose, 23-nordeoxycholic acid, (±)18-HEPE, (±)15-HEPE, (±)12-HEPE, and acetaminophen sulfate were significantly decreased (Figure 8C). The identified biomarkers distinguishing the M and PO groups were primarily associated with pathways involved in digestion and secretion functions, signal transduction and regulation, metabolism and biosynthesis, and disease-related pathways (Figure 8F).

In the comparison between the PO group and the HOPO group, five differential metabolites were identified: hexanoyl glycine, pantothenol, 9,10-DiHOME, 2′-O-methylcytidine, and 12,13-DiHOME. The levels of these differential metabolites were significantly lower in the HOPO group compared to the PO group (Figure 8D). The identified biomarkers distinguishing the PO and HOPO groups were primarily associated with pathways involved in linoleic acid metabolism, and pantothenate and CoA biosynthesis (Figure 8H).

## 4. Discussion

The human gut contains over 100 trillion microorganisms, including bacteria, archaea, viruses, and fungi, collectively referred to as the gut microbiome [25]. Although mycobiomes make up only about 0.1% of the gut microbiome, they are a diverse and essential component, contributing to intestinal homeostasis and disease pathogenesis [26,27]. The gut microbiome may play an important role in metabolic health by influencing nutrient absorption, interacting with other microorganisms, regulating inflammatory responses, and being affected by diet [28]. Understanding these mechanisms can help develop new intervention strategies to improve metabolic health. Much research has investigated the effects of dietary fatty acid composition on gut bacteria and metabolic health, with particular emphasis on dietary fats rich in oleic acid [29,30,31,32], as this is a key characteristic of the Mediterranean diet. Our previous study revealed that HOPO is more effective than regular peanut oil in alleviating insulin resistance caused by a HFFD, likely through the modulation of gut bacterial structure and suppression of proteolytic fermentation in the distal colon [24]. However, the impact of dietary fatty acid composition on the structure and function of the gut mycobiome remains poorly understood.

In this study, we found that the oleic acid content of dietary fat also has a significant impact on the composition and metabolic characteristics of gut mycobiome in rats fed a HFFD. The α-diversity analysis showed that both HOPO and PO reversed the reduction in gut fungal diversity induced by the HFFD. The α-diversity of gut mycobiome in the PO group was significantly higher than that in the HOPO group. However, in our previous study on gut bacteria, the opposite result was observed, with the HOPO group showing higher bacterial diversity [24]. We hypothesize that under specific dietary influences, there is a certain antagonistic relationship between gut fungal diversity and gut bacterial diversity. It has been demonstrated that the growth of *Lactobacilli* competes with fungi and may inhibit the expansion of *Candida* [33]. To date, variations in the impact of gut fungal α-diversity across different diseases or even among subtypes of the same disease contribute to the difficulty in reaching consistent conclusions [34,35,36,37]. The human and mammalian gut harbors commensal fungi, with *Ascomycota* and *Basidiomycota* as the dominant fungal phyla [38]. This is consistent with the findings of this study. Moreover, both the *Basidiomycota*/*Ascomycota* ratio and the fungal diversity/bacterial diversity ratio may be linked to gut homeostasis. A significant increase in these ratios has been observed in patients with inflammatory bowel disease, potentially indicating an imbalance in the gut microbiome. The imbalance in the Basidiomycota/Ascomycota ratio is closely associated with the functional deficiency of the host Card9 and Dectin1 genes, which play a crucial role in maintaining gut microbiota balance and immune homeostasis [34]. However, the causal relationship behind this association still needs further clarification. In this study, the effects of HFFD on the *Basidiomycota*/*Ascomycota* ratio in the gut fungi of rats were different. The *Basidiomycota*/*Ascomycota* ratio decreased in both the M and HOPO groups compared to the NC group (NC: 0.012, M: 0.0034, HOPO: 0.0089), whereas it increased in the PO group (PO: 0.11). Based on our previously published gut bacterial data, the fungal diversity/bacterial diversity ratio (calculated based on OTU counts) in each group of rats showed that the HOPO group was the closest to the NC group (HOPO: 0.24, NC: 0.26), while the M group and PO group exhibited a marked decrease and increase compared to the NC group (M: 0.15, PO: 0.39), respectively. Therefore, we speculate that the fungal diversity/bacterial diversity ratio is a more effective indicator of diet-induced gut microbiome dysbiosis, with both excessively low and high ratios representing an imbalance of gut microbiome.

Dietary pattern factors affecting the composition of gut microbiome in individuals commonly influence fungi belonging to the phyla *Ascomycota* and *Basidiomycota* [38]. This is also consistent with the findings of this study. LEfSe analysis revealed four biomarkers (*Ascomycota*, *Saccharomycetales*, *Saccharomycetes*, and *Sordariomycetes*), all classified under *Ascomycota*, displaying significant differences between groups. *Ascomycota* is an important component of the gut microbiome, with common genera including *Saccharomyces*, *Candida*, *Cladosporium*, and *Penicillium* [39,40]. In overweight and obese individuals, the abundance of *Basidiomycota* increases. Certain fungi from the *Basidiomycota* phylum, such as *Malassezia*, may be associated with fat metabolism and inflammatory responses, thereby influencing overall metabolic health [41]. In this study, it was also observed that *Basidiomycota* was increased in the PO group compared to the HOPO group, but the abundance of *Basidiomycota* was very low in the M group. Therefore, the metabolic health of rats cannot be simply attributed to the increased abundance of *Basidiomycota*. At the genus level, the seven fungal taxa identified as connectors in fungal interaction networks—*Aspergillus*, *unclassified_Basidiomycota*, *Alternaria*, *Vishniacozyma*, *Penicillium*, *Pseudogymnoascus*, and *Thermothielavioides*—belong to the phyla *Ascomycota* and *Basidiomycota*. *Aspergillus* releases mycotoxins into non-neuronal tissues, which can subsequently enter the bloodstream [42]. When present in high abundance, it becomes pathogenic and poses a significant risk to immunocompromised individuals [43,44]. An increased abundance of *Aspergillus* and *Penicillium* has been suggested to be associated with the obese phenotype [45]. In this study, compared to the NC group, the abundance of *Aspergillus* and *Penicillium* decreased in the HOPO and M groups of rats, while it increased in the PO group. *Candida*, a common gut fungal genera, has been increasingly linked to metabolic diseases. Studies suggest that an overgrowth of *Candida* can disrupt the gut microbial balance, leading to inflammation and metabolic dysregulation. For instance, *C. albicans* has been associated with obesity, insulin resistance, and type 2 diabetes, potentially through mechanisms such as promoting gut permeability and triggering systemic inflammation [46,47,48]. The abundance of *Candida* was higher in the HFFD group than in the NC group. However, this trend was reversed exclusively by HOPO. This effect may be attributed to HOPO’s superior ability to mitigate HFFD-induced insulin resistance in rats.

In terms of serum metabolomics in rats, HFFD induced an increase in 23-nordeoxycholic acid, while this trend was reversed by HOPO and PO. Bile acids are synthesized in the liver and converted by the gut microbiota into secondary bile acids, which are closely associated with fat metabolism. For example, secondary bile acids, such as 23-nordeoxycholic acid can serve as a biomarker for bile acid metabolism, gut microbiota balance, and overall metabolic health [49]. Disruption of the bile acid metabolic pathway may lead to reduced insulin sensitivity through the FXR and the TGR5 [50]. In contrast, HFFD significantly reduced the serum taurochenodesoxycholic acid levels in rats, while HOPO and PO increased the serum taurochenodesoxycholic acid levels. Compared to the PO group, the HOPO group showed a greater increase, bringing the levels closer to those of the NC group. Therefore, altered serum levels of 23-nordeoxycholic acid and taurochenodesoxycholic acid may reflect disruptions in bile acid metabolism and imbalances in the gut microbiota. Additionally, compared to the NC group, pantothenol levels were elevated in both the M and PO groups, but this increase was exclusively reversed by HOPO. Pantothenol is a precursor of vitamin B5 (pantothenic acid). In the body, it is converted into pantothenic acid, which plays a key role in the synthesis of coenzyme A (CoA). CoA is involved in energy metabolism, fatty acid synthesis, and breakdown, as well as important physiological processes such as cell repair [51,52]. Elevated serum pantothenol levels may indicate physiological abnormalities such as impaired CoA synthesis or metabolism. Based on previous studies showing that HOPO has a better effect than PO in alleviating HFFD-induced glucose and lipid metabolism disorders [24], maintaining normal CoA metabolism may be its potential mechanism of action. The results of KEGG pathway enrichment analysis based on differential metabolites between groups also support this hypothesis. The pantothenate and CoA biosynthesis pathway was enriched in both the HOPO vs. M group and the HOPO vs. PO group.

This study enhances the understanding of the effects of dietary fatty acid composition on the gut mycobiome and blood metabolome in rats. However, several limitations should be acknowledged. First, the gut mycobiome exhibits high heterogeneity, especially in small-sample animal studies. Therefore, in future research, it would be helpful to strictly control the source of animals, conduct fungal analysis before and after dietary interventions, and validate the findings in large-sample and multi-center cohort studies. Second, this study did not investigate potential molecular processes, including the regulation of glucose and lipid metabolism pathways. Future research needs to focus on elucidating these mechanisms in greater detail and conducting dietary intervention experiments specifically targeting patients with nutritional and metabolic disorders.

## 5. Conclusions

To summarize, this study highlights the significant impact of dietary fatty acid composition, particularly oleic acid-rich oils, on the gut mycobiome and blood metabolome in HFFD-fed SD rats. Both HOPO and PO were effective in reversing HFFD-induced reductions in gut fungal diversity, with HOPO demonstrating superior effects in restoring gut microbiome balance, as indicated by the fungal diversity/bacterial diversity ratio and reduced abundance of pathogenic fungi such as *Candida*. Additionally, HOPO showed a greater ability to normalize serum bile acid levels, including taurochenodesoxycholic acid, and reverse elevated pantothenol levels, suggesting its potential role in maintaining bile acid metabolism and CoA biosynthesis. However, further studies are needed to validate these findings in humans and to explore the underlying molecular mechanisms in detail.

## Figures and Tables

**Table 1:** Nutrient profiles of the test diets.

	NC	M	HOPO	PO
Caloric ratio/% kcal				
Fat	10	45	45	45
Protein	20	20	20	20
Carbohydrate	70	35	35	35
Fat source/% weight				
HOPO	—	—	11.8	—
PO	—	—	—	11.8
Soybean oil	2.4	2.9	2.9	2.9
Lard	1.9	20.7	8.9	8.9
Others/% weight				
Casein	19.0	23.3	23.3	23.3
L-Cystine	0.3	0.3	0.3	0.3
Corn Starch	42.9	8.5	8.5	8.5
Maltodextrin	7.1	11.7	11.7	11.7
Sucrose	16.8	20.6	20.6	20.6
Cellulose	4.7	5.8	5.8	5.8
Mineral Mix	4.7	5.8	5.8	5.8
Vitamin Mix	0.1	0.1	0.1	0.1
Choline Bitartrate	0.2	0.2	0.2	0.2

## Data Availability

The original contributions presented in this study are included in the article/Supplementary Materials, and further inquiries can be directed to the corresponding author.

## Supplementary Materials

The following supporting information can be downloaded at: https://www.mdpi.com/article/10.3390/foods14030506/s1, Figure S1: permutation tests for validation of OPLS-DA models.
