# Supplementary material for: Modulation of Gut Mycobiome and Serum Metabolome by a MUFA-Rich Diet in Sprague Dawley Rats Fed a High-Fructose, High-Fat Diet
Source: Foods. 2025 Feb 5;14(3):506. doi: 10.3390/foods14030506 (PMC11816726; doi:10.3390/foods14030506)
Supplement: Supplementary file 1 [file foods-14-00506-s001.zip › foods-3455588-supplementary.pdf]

A

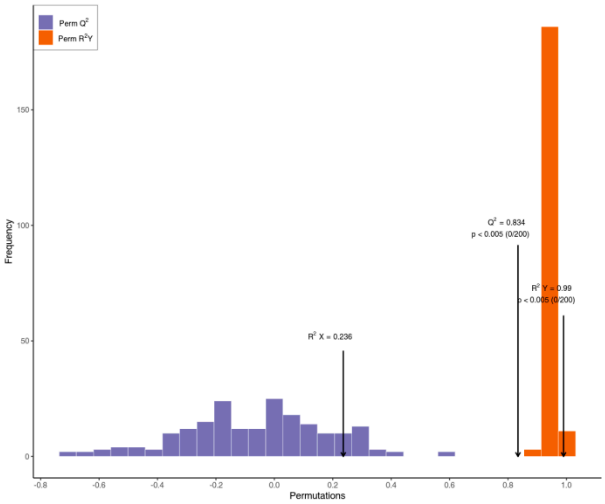
Perm Q^2
Perm R^2Y
Frequency
Permutations
Q^2 = 0.834
p < 0.005 (0/200)
R^2 Y = 0.99
p < 0.005 (0/200)
R^2 X = 0.236

B

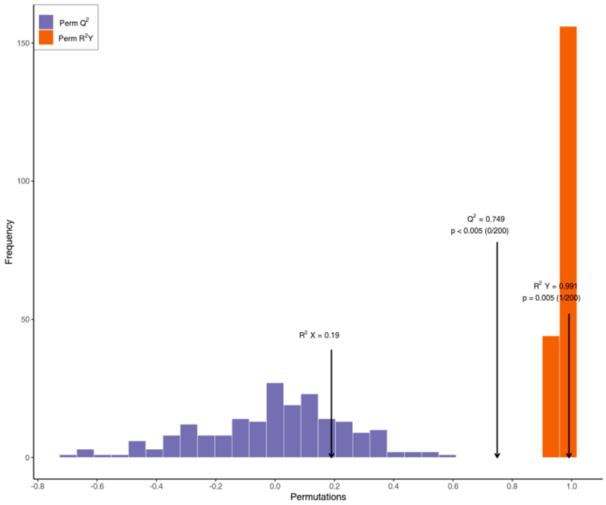
Perm Q^2
Perm R^2Y
Frequency
Permutations
Q^2 = 0.749
p < 0.005 (0/200)
R^2 Y = 0.991
p = 0.005 (1/200)
R^2 X = 0.19

C

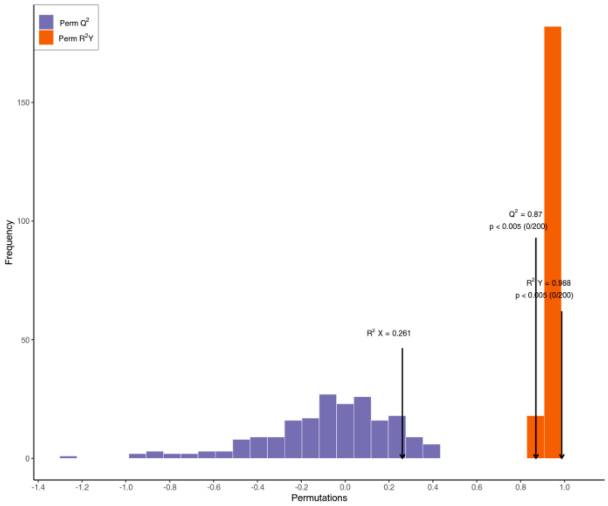
Perm Q^2
Perm R^2Y
Frequency
Permutations
Q^2 = 0.87
p < 0.005 (0/200)
R^2 Y = 0.988
p < 0.005 (0/200)
R^2 X = 0.261

D

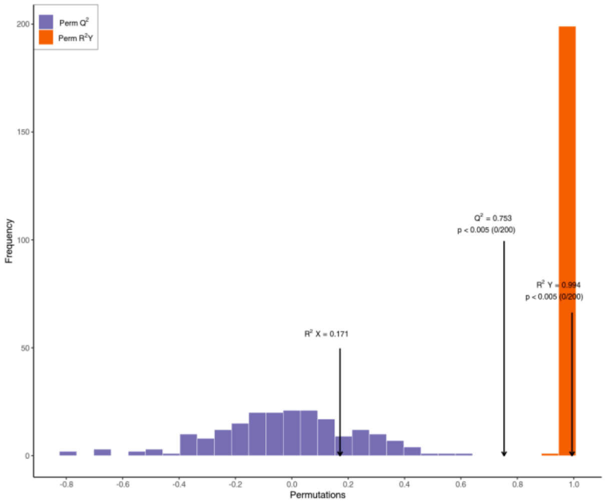

Figure S1. Permutation tests for validation of OPLS-DA models: (**A**) between NC and M groups; (**B**) between M and HOPO groups; (**C**) between M and HOPO groups; (**D**) between PO and HOPO groups.
